# An Analysis of the Eurasian Tectonic Plate Motion Parameters Based on GNSS Stations Positions in ITRF2014

**DOI:** 10.3390/s20216065

**Published:** 2020-10-25

**Authors:** Marcin Jagoda, Miłosława Rutkowska

**Affiliations:** Faculty of Civil Engineering Environmental and Geodetic Sciences, Koszalin University of Technology, Śniadeckich 2, 75-453 Koszalin, Poland; miloslawa.rutkowska@tu.koszalin.pl

**Keywords:** GNSS stations, tectonic plate motion parameters, Eurasian plate, ITRF

## Abstract

The article is the fourth part of our research program concerning an analysis of tectonic plates’ motion parameters that is based on an observation campaign of an array of satellite techniques: SLR, DORIS, VLBI, and now GNSS. In this paper, based on the International Terrestrial Reference Frame 2014 (ITRF2014) for observations and using the GNSS technique, the Eurasian tectonic plate motion was analyzed and the plate motion parameters Φ, Λ (the position of the rotation pole), and *ω* (the angular rotation speed) were adjusted. Approximately 1000 station positions and velocities globally were obtained from the GNSS campaign over a 21-year time interval and used in ITRF2014. Due to the large number of data generated using this technique, the analyses were conducted separately for each tectonic plate. These baseline data were divided into a number of parts related to the Eurasian plate, and are shown in this paper. The tectonic plate model was analyzed on the basis of approximately 130 GNSS station positions. A large number of estimated station positions allowed a detailed study to be undertaken. Stations that agree with the plate motion were selected and plate parameters were estimated with high accuracy. In addition, stations which did not agree with the tectonic plate motion were identified and removed. In the current paper, the influence of the number and location of stations on the computed values and accuracy of the tectonic plate motion parameters is discussed. Four calculation scenarios are examined. Each scenario contains 30 stations for the common solution of the European and Asiatic part of the Eurasian plate. The maximum difference between the four calculation scenarios is 0.31° for the Φ parameter and 0.24° for the Λ parameter, indicating that it is at the level of the value of the formal error. The ω parameter has the same value for all the scenarios. The final stage of the analysis is the estimation of parameters Φ, Λ, and *ω* based on all of the 120 stations used in the four calculation scenarios (i.e., scenario 1 + scenario 2 + scenario 3 + scenario 4). The following results are obtained: Φ = 54.81° ± 0.37°, Λ = 261.04° ± 0.48°, and *ω* = 0.2585°/Ma ± 0.0025°/Ma. The results of the analysis are compared with the APKIM2005 model and another solution based on the GNSS technique, and a good agreement is found.

## 1. Introduction

The earth, due to its dynamics, requires constant observation. Continuous changes occurring inside the earth caused by various geophysical and geological phenomena are reflected on the surface of the globe [1], such as in the form of tectonic plate movements. The theory of the movement of tectonic plates (the so-called continental drift hypothesis) was first published in 1915 by A. Wegener in his fundamental work "Die Entstehung der Kontinente und Ozeane" [2]. In the following years of the 20th century a number of scholars addressed these issues, e.g., geologists O.C. Hilgenberg [3], F.A. Vening-Meinesz [4], S.W. Carey [5], and X. Le Pichon [6], until the adoption by the scientific community in 1968 of the so-called Theory of Plate Tectonics, whose assumptions were described in [7]. 

During the 1980’s and 1990’s of the last century a dynamic development of satellite observation techniques (GPS, SLR, DORIS, VLBI) occurred, providing an unprecedented opportunity to conduct research on a global scale with very high accuracy. These techniques were also applied in the study of tectonic plate motion. The accuracy of determining the position of points on the earth’s surface using satellite methods has continuously increased, and this increase in accuracy has allowed the numerical values describing the movement of tectonic plates to be determined. Continuity of research in this field is a noticeable trend, e.g., GNSS technique: [8,9,10,11,12]; SLR technique: [13,14,15,16,17,18]; DORIS technique: [10,19,20]; and VLBI technique: [21,22,23,24,25,26].

Initially, geological methods based on the description of the mechanism of tectonic plate motion were used to study the movement of tectonic plates; only the satellite techniques mentioned above made it possible to precisely estimate the numerical values of this movement, as discussed in [27]. Thorough knowledge and the ability to mathematically describe the phenomena causing the movement of the tectonic plates, and the possibility of taking precise measurements using satellite techniques, allows points on the earth’s surface and their annual movements to be determined with high accuracy. This is the basis for determining the parameters that describe the movement of tectonic plates. Tectonic plates move in relation to each other on the astenospheric surface at a speed ranging from a few to several centimeters per year. The lithosphere is constantly destroyed in the subduction zones and is renewed on mid-ocean ridges. Continents usually form a section of a plate and are moved with it. The following types of boundaries between the plates exist: divergent, convergent, and horizontally sliding. Issues related to these boundaries have been previously addressed, e.g., [28,29]. Large stresses can be present on the edges of the plates. These are discharged in the form of earthquakes. In the case of tectonic plates, it is difficult to speak of rigid boundaries between plates and to indicate where one plate ends and another begins; at the plate boundaries, there is often an area in which displacements are incompatible with the movement of the whole plate.

The movement of the tectonic plate is described by the rotation vector Ω, whereas the parameters of this movement are described by the geographical position of the rotation pole—Φ and Λ—and the angular velocity of rotation—*ω* (or by individual components of the rotation pole) [30,31]. According to [30], the displacement of the observational station as a function of the parameters of plate motion is expressed by Equation (1):

(1) $$\begin{array}{c} \Delta \phi =\omega \cdot \Delta t\cdot \cos\Phi \cdot \sin(\lambda -\Lambda )\\ \Delta \lambda =\omega \cdot \Delta t\cdot (\sin\Phi -\cos(\lambda -\Lambda )\tan\phi \cdot \cos\Phi ), \end{array}$$

where:

Δ*ϕ*, Δ*λ*—displacement of the observational station position in latitude and longitude;
*ϕ*, *λ*—observational station position (GNSS, SLR, DORIS, VLBI);
Φ, Λ, *ω*—plate motion parameters (the position of the rotation pole in latitude and longitude, and the angular rotation speed, respectively).

The aim of this study is to estimate and analyze the parameters describing the motion of the Eurasian plate based on GNSS station positions, and to identify and eliminate from the calculation those stations whose motion is not consistent with the motion of the plate, hence “polluting” the results of the motion parameters and increasing the value of the formal error.

## 2. Materials and Methods

There are approximately 1000 GNSS stations located around the world whose positions are provided in the International Terrestrial Reference Frame 2014 (ITRF2014) [32]. From this baseline data, about 130 stations located on the Eurasian continent were analyzed. These stations are not uniformly distributed. Approximately 70% of the stations are located in the European part of the Eurasian plate. Generally, however, the number of estimated station positions and velocities is sufficiently large, and allows plate motion parameters to be estimated with high accuracy and in detail. It also allows stations that are not compatible with the Eurasian tectonic plate motion to be identified and removed, and only stations that are consistent with the solution to be selected. 

**Figure 1:**
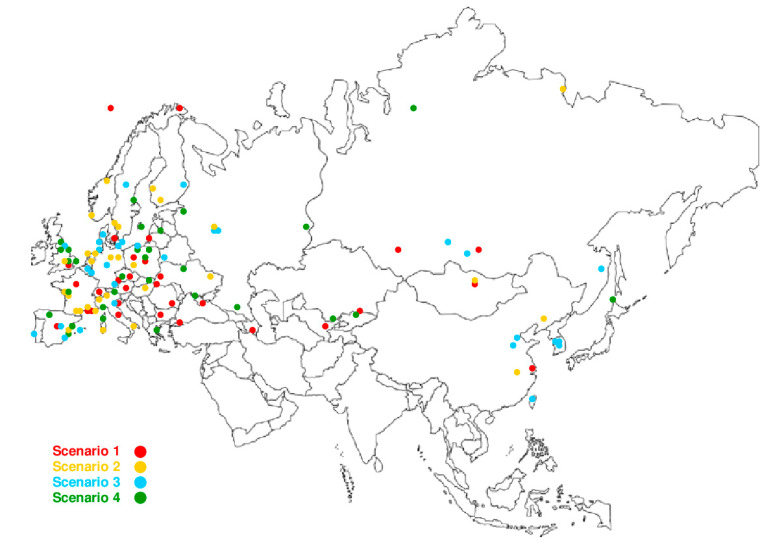
The location of the GNSS stations used in the four scenarios of the calculations.

**Figure 2:**
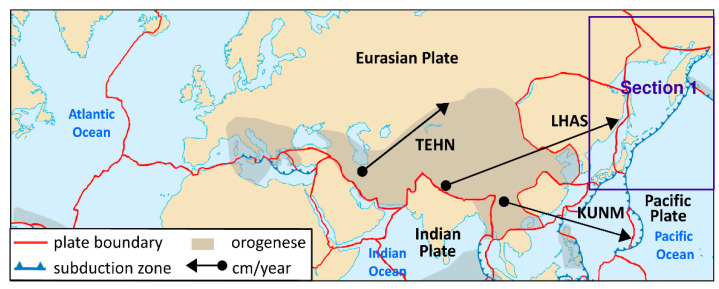
The location and motion of the GNSS stations that are not consistent with the Eurasian plate motion.

**Figure 3:**
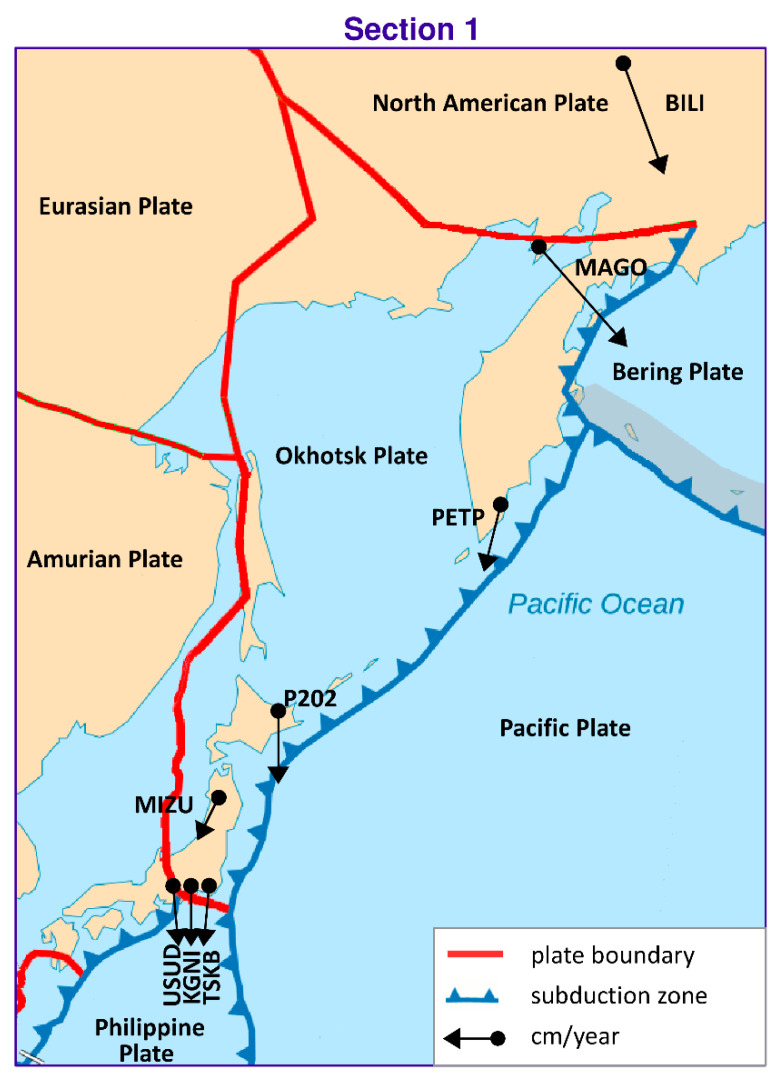
The location and motion of the GNSS stations that are not consistent with the Eurasian plate motion; area of Section 1 (Figure 2).

The baseline data were divided into four calculation scenarios. Each scenario contained 30 randomly distributed stations for a common solution of the Eurasian plate. The stations used in the four scenarios of the calculations are tabulated in Appendix A, Appendix B, Appendix C, Appendix D, separately for each scenario, and are shown in Figure 1. Detailed information on individual stations, including their positions and velocities, is presented on the ITRF website: http://itrf.ensg.ign.fr/ITRF_solutions/2014/. The final solution of the calculation was determined from all of the 120 stations (scenario 1 + scenario 2 + scenario 3 + scenario 4). The locations of the GNSS stations that are not consistent with the Eurasian plate motion are shown in Figure 2 and Figure 3. All calculations were carried out using the authors’ own software.

The description of the method applied in this paper to determine the plate motion parameters, and the theoretical basis for this motion, has been extensively presented by the authors in their earlier papers on the analysis of the plate motion based on movements of observation stations: SLR [33,34]; DORIS [34,35]; and VLBI [34,36]. Thus these aspects were omitted herein.

## 3. Tectonic Plate Theory for the Eurasian Plate

The Eurasian plate, which is the subject of this analysis, is the most complex part of the earth in terms of geological structure [37]. On the western, northern, and north-eastern sides, the plate borders the North American plate. In the west and north, this boundary is a divergent one, on which the Mid-Atlantic Ridge was formed. In the east it is a convergent boundary [38]. In the north, the area of Sweden and southern Norway has been subject to continuous uplift movements for about 1.5 billion years to date [39]. The north-eastern part of the Asian continent belongs to the North American plate, whereas the area comprising the Koryak Mountains and the Kamchatka area belongs to the Eurasian plate; however, the course of the boundary itself has not yet been sufficiently studied. The Eurasian plate also borders on the Bering and Pacific plates, which are being pulled under the Eurasian plate, constituting is a convergent boundary. Two small plates are positioned between the Pacific and Eurasian plates: the Okhotsk plate, including the island of Sakhalin on which tectonic movements are ongoing, and the Amur plate. These plates are often treated as part of the Eurasian plate because their motion is similar to that of the latter plate [40]. A zone of islands with heterogeneous crust structure extends from Hokkaido in the north to Taiwan in the south. This string lies at the meeting point of the Pacific, Philippine, Okhotsk, and Amur plates. The boundary between the Philippine and Pacific plates is a convergent one, running through the island of Honshu. In Indonesia, eastern Asia joins the Pacific range. The southern boundary of the Eurasian plate with the African, Anatolian, Arabic, and Indo-Australian plates is defined by approximately 12,000 km of the Alpine-Himalayan range, the Alpine fold zone connecting through the Strait of Gibraltar with the Alpine structures of the Atlas mountains, and the Alpine ranges in Asia. This boundary includes the Atlas, the Pyrenees, the Alps, the Apennines, the Carpathians, the Rhodopes, the Caucasus, the Tien Shan, Tibet, the Himalayas as far as Burma, and the Andaman Sea. The Apennines were created in the process of subduing the Tyrrhenian and Adriatic plates. The Caucasus was created as a result of a collision between the Anatolian and European plates. The area is characterized by high seismicity and is still subject to tectonic processes. In this region, the plates overlap and are squeezed and folded, the continents collide, and the phenomenon of orogenesis takes place. Now, this phenomenon occurs at the place where the Indian and Eurasian plates meet. This collision began about 40 million years ago. Since that contact, the Indian Peninsula has moved 2000 km into Eurasia. The consequences of this collision include the creation of the Himalayas, Tibet, and Tien Shan, in addition to earthquakes in China and north-western Iran [41,42]. The processes associated with the formation of the continental structure continue to this day. The Indian Peninsula moves northwards at a speed of 3.3–4.8 cm per year, resulting in a continuous uplift of the Himalayas. The Arabian Peninsula moves to the northeast at a speed of 1.4–1.8 cm per year. The Pacific plate is subdued by the Asian continental lithosphere at a speed of 6.7–7 cm per year. These processes result in strong earthquakes, tsunamis, and volcanic eruptions [43].

For the Eurasian, African, and Arabic plates, the whole area of southern Europe and the Mediterranean area is considered a boundary zone. The lithosphere here is very cracked and forms a large number of micro plates [44]. The African plate is rubbing against the Eurasian and Arabic plates. The area is highly seismically active and the lithosphere is strongly cracked and divided into the Anatolian, Black Sea, Aegean, Macedonian, Adriatic, Corsican, Atlas, and Iberian micro plates. The north-eastern part of Corsica belongs to the Alpine structures and came into existence as a result of collisions with the micro plate of the Tyrrhenian Sea (the Apulian plate) and the African plate.

## 4. Results and Discussion

**Figure 4:**
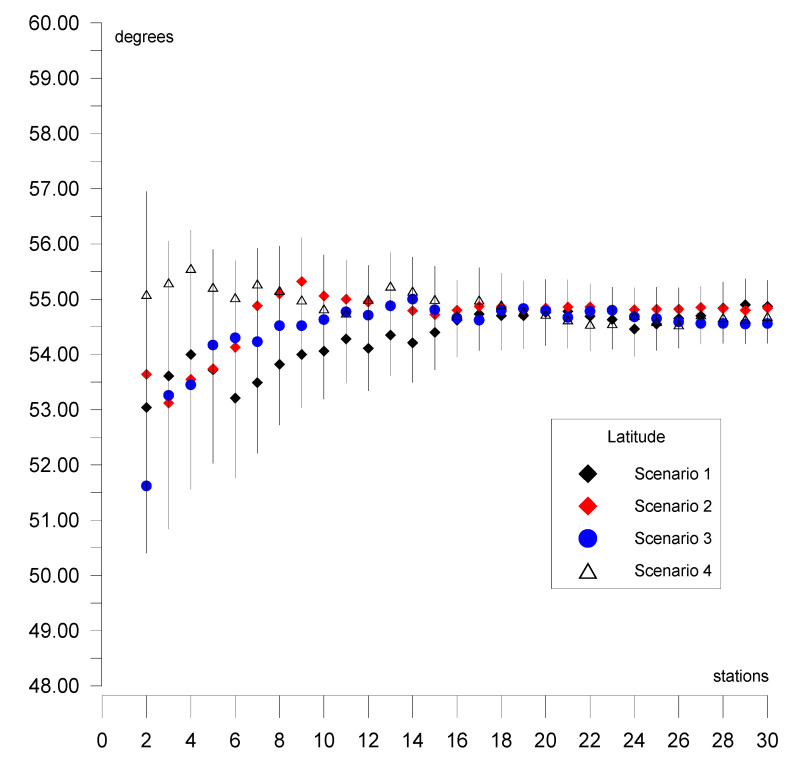
Results of the sequential solutions of the Φ parameter for four calculation scenarios.

**Figure 5:**
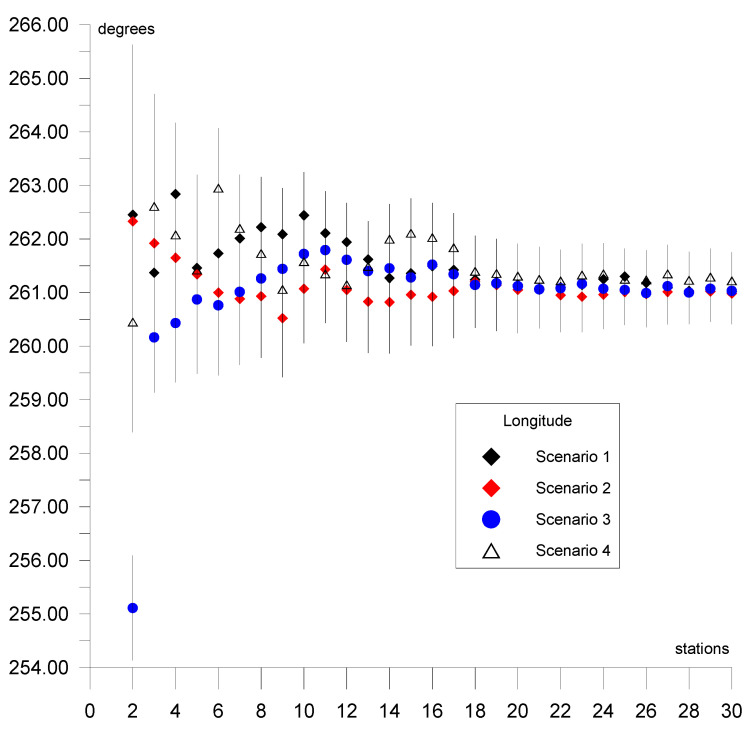
Results of the sequential solutions of the Λ parameter for four calculation scenarios.

**Figure 6:**
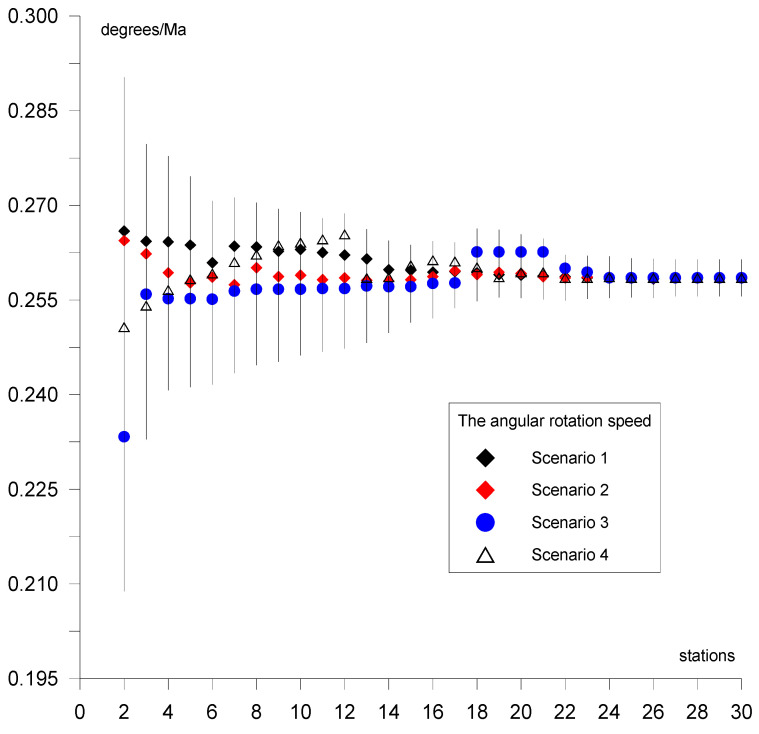
Results of the sequential solutions of the *ω* parameter for four calculation scenarios.

This section presents the results of the estimation of the Eurasian tectonic plate motion parameters based on GNSS station positions in ITRF2014 [32]. Initially, the solution was derived for two stations located on a plate. Then, single stations were added individually (sequential solution) for a maximum of 29 steps for 30 stations (in each of the four scenarios of calculations). The selection of the stations in the scenarios was based on the following criterion: first stations located in the stable region of the plate were included; then, stations located near the plate boundary but which did not disturb the solution accuracy were included. Results for the four calculation scenarios are given in Figure 4, Figure 5 and Figure 6 and are summarized in tables (Appendix A, Appendix B, Appendix C, Appendix D) for values of plate motion parameters Φ, Λ, and ω. The stability of the parameters and their errors for scenarios 1–4 is observed for about 18–20 stations. For the subsequently added stations, the estimated plate parameter values differ by less than the value of the formal error. This proves that the necessary number of stations is about 20. The final solutions based on 30 stations for the four calculation scenarios are as follows: parameter Φ for scenario 1 equals 54.87° ± 0.47°, for scenario 2 equals 54.84° ± 0.38°, for scenario 3 equals 54.56° ± 0.36°, and for scenario 4 equals 54.69° ± 0.41°. The maximum difference amounts to 0.31°, which is consistent with the level of the value of the formal error. Parameter Λ for scenario 1 equals 261.02° ± 0.50°, for scenario 2 equals 260.98° ± 0.57°, for scenario 3 equals 261.03° ± 0.46°, and for scenario 4 equals 261.22° ± 0.53°. The maximum difference between the scenarios amounts to 0.24°, which is less that the value of the formal error. Similarly, for ω the result equals 0.2585°/Ma for all of the scenarios. This indicates the high consistency of these solutions after elimination of erroneous stations. First, erroneous stations and those not compatible with the Eurasian plate motion or not located on the Eurasian plate were identified and deleted from the analysis. The analysis concerning selection of these stations was performed for calculation scenario 1 (given in Appendix A) and is presented in Table 1. The second line of this table shows the result of the final solution for calculation scenario 1. Next, “doubtful” stations were analyzed. Lines 3 to 13 of Table 1 provide the results obtained after the addition of 30 individually selected “doubtful” stations given in scenario 1 and the new solution resulting from 31 stations. Adding Bilibino (BILI) station located on the North American plate on which part of the Asian continent is situated (geodetic position is approx. B = 67°, L = 166°) to the solution changes the plate parameters by about 1.8° and 1.4° in latitude and longitude, respectively. This station should not be included in the solution because differences are greater than the values of error. Next, Magadan (MAGO) station is located on the boundary of two plates, namely, the North American plate and the small Okhotsk plates. The geodetic position of the MAGO station is equal to approx. B = 59°, L = 150°. This station changes the plate parameters by about 1.2° and 1.3° in latitude and longitude, respectively. Differences are greater than the values of error, therefore this station should not be included in the solution. Station Petropavlovsk (PETP) is located on the Kamchatka Peninsula, and its geodetic position is equal to approx. B = 52°, L= 158°. This station changes the plate parameters significantly, by about 4.5° and 6.8° in latitude and longitude, respectively. The differences are about 9 times greater than the values of error; hence, this station is eliminated from the solution. Teheran (TEHN) station is located near the boundary of the Iran plate in the geodetic position of approx. B = 35°, L = 51°. This station changes plate parameters insignificantly, by about 0.2° and 0.4° in latitude and longitude, respectively, but also increases the values of error of the determined parameters and is not be included in the solution. Next, Kunming (KUNM) station is located in South Himalaya in a very seismically active region of China on the Eurasian plate, and has a geodetic position of approx. B = 24°, L = 102°. The station changes plate parameters by about 0.8° and 1° in latitude and longitude, respectively, and is not used in the solution. Lhasa (LHAS) station is located on the boundary of the Eurasian plate and the Deccan plate in South Himalaya, and its geodetic position is approx. B = 29°, L = 91°. The station significantly increases the error of the plate parameters, by about 2.8° and 4° in latitude and longitude, respectively. Due to these values this station is eliminated from the solution. The Japanese stations of Mizusawa (MIZU), Koganei (KGNI), Usuda (USUD), Tsukuba (TSKB), Mitaka (MTKA), and Abashiri (P202) are located at B = 35–44°, L = 138–144° near the boundary of the Pacific, Okhotsk, Amur, and Philippine plates. Shifts of these stations are not consistent with the Eurasian plate motion and values of motion vectors are significantly smaller, thus, these stations are not included in the solution.

The stations with velocity directions and values that are not consistent with the Eurasian plate motion are not included in the solution (these are shown in Figure 2 and Figure 3). Inclusion of these stations results in a description of the Eurasian plate motion that is incompatible with the real movement of the whole plate rotation around poles Φ and Λ.

The final stage of the analysis was the estimation of parameters Φ, Λ, and *ω* based on all of the 120 stations used in the four calculation scenarios (scenario 1 + scenario 2 + scenario 3 + scenario 4). The following results were obtained: Φ = 54.81° ± 0.37°, Λ = 261.04° ± 0.48°, and *ω* = 0.2585°/Ma ± 0.0025°/Ma. These results were compared with the APKIM2005 model by H. Drewes [45] and with another solution based on the GNSS technique given in Larson et al. [8]. The comparison is shown in Table 2. The APKIM2005 model [45] is based on weekly solutions for SLR, DORIS, VLBI, and GNSS techniques observed for the time interval from 1993 to 2004. The database for these techniques allows estimation of the velocity vectors caused by plate motion and plate motion parameters for the following 17 plates: African, Amur, Antarctic, Arabian, Anatolian, Australian, Caribbean, Eurasian, Indian, Nazca, North American, South American, Okhotsk, Pacific, Somalia, Sunda, and Yangtze. In [8] authors analyzed the GNSS data from January 1991 to March 1996. All of the presented data were analyzed using the GIPSY/OASIS II software. On the basis of the computed network, velocities for 38 sites located on the African, Antarctic, Australian, North American, South American, Pacific, and Eurasian plates were estimated. For the Eurasian plate, the parameters were estimated on the basis of eight stations; six of these are located on the European part of the plate, and two are located on the Asian plate.

Plate motion parameters obtained by Drewes for the Eurasian plate equal: Φ = 53.4° ± 0.4°, Λ = 264.3° ± 0.5°, and *ω* = 0.259°/Ma ± 0.001°/Ma [45]. However, in Larson’s determination [8] they are: Φ = 56.3°, Λ = 257.2°, and *ω* = 0.26°/Ma. The differences between these three solutions are approximately 3 and 7° for Φ and Λ, respectively, whereas for *ω* parameter the difference amounts to 0.0015°/Ma.

## 5. Conclusions

The computations and results of the analysis given in this paper allow the following conclusions to be drawn:

- The plate motion parameters for the final solution based on 120 (scenario 1 + scenario 2 + scenario 3 + scenario 4) GNSS station positions taken from ITRF2014 are equal to Φ = 54.81° ± 0.37° for latitude, Λ = 261.04° ± 0.48° for longitude, and *ω* = 0.2585°/Ma ± 0.0025°/Ma for rotation speed.
- The convergence and stability of the solutions for the four calculation scenarios based on 30 randomly distributed stations for each scenario are obtained for about 18–20 stations, as presented in Figure 4 for latitude Φ, Figure 5 for longitude Λ, and Figure 6 for rotation speed *ω*.
- The selection of suitable stations for determining the parameters allows the determination on the basis of approximately 20 stations to be made, as shown in the four calculation scenarios. Adding more stations to the calculations results in a change in the value of the determined parameters by a value that does not exceed the formal error.
- The geological model of the Eurasian plate is the most complicated on earth. The Eurasian plate includes a subduction zone in the convergent eastern boundary. Part of the North American plate, on which Bilibino (BILI) station is situated, is located in the eastern section of the Eurasian continent. The annual shift of this station is not consistent with the Eurasian plate motion. This station cannot be included in the solution because of a change in the pole rotation of the plate parameters by approx. 2 degrees in latitude, which corresponds to 200 km, and 1.5 degrees in longitude, which corresponds to 150 km. Magadan (MAGO) station is located on the boundary of the American and Okhotsk plates. The annual shift of the station is not consistent with the Eurasian plate motion by approx. 1 degree in latitude, which equals 100 km, and 1 degree in longitude, which also equals 100 km, therefore this station cannot be used in the solution. Petropavlovsk (PETP) station is located on the Okhotsk plate on Kamchatka Peninsula. The shift of this station is also not consistent with the Eurasian plate motion, and amounts to 4.5 degrees in latitude, which corresponds to 450 km, and 6.8 degrees in longitude, which equals 680 km, hence the station cannot be used in the solution. Teheran (TEHN) station is also not used in the solution because it is located near the boundary of the Iran plate in a high seismic activity region, despite a relatively small change in the pole rotation of plate parameters, by approx. 0.2 degrees in latitude, which corresponds to 20 km, and 0.4 degrees in longitude, which equals 40 km.
- The Eurasian plate contains areas of high seismic activity and cracked boundaries, both convergent and divergent, with other tectonic plates, such as the Pacific, Deccan, Iran, Arabian, and Anatolian plates. Often, shifts of the analyzed stations located on the Eurasian plate are not compatible with the tectonic plate motion; for example, Lhasa (LHAS) station, which is located on the boundary of the Eurasian plate and the Deccan plate in the South Tibet and Himalaya mountains, is in a very active and cracked region. This station increases the error of the estimated plate parameters significantly, by approx. 3 degrees in latitude, equaling 300 km, and 4 degrees in longitude, equaling 400 km. The station is not included in the solution.
- The Japanese stations of Mizusawa (MIZU), Koganei (KGNI), Usuda (USUD), Tsukuba (TSKB), and Abashiri (P202) are located in a very active and cracked area on the boundary of the Philippine plate. In this region, three cracked plates come into contact: the Pacific plate with the Okhotsk and Amur plates. Shifts of these stations are not consistent with the Eurasian plate motion and are not included in the solution.
- Wakkanai station (used in calculation scenario 4) is located on the boundary of the Amur and Okhotsk plates. The shift of this station is consistent with the Eurasian plate motion to a high degree, therefore it is included in the solution.
- The application of the sequential calculation method allows stations whose movement is not consistent with that of the entire plate to be identified and eliminated from the solution.
- Analysis and elimination of the selected stations, as shown in Table 1, is indispensable because it allows results to be obtained only on the basis of the stations with shifts that are consistent with the Eurasian plate.
- The Mediterranean Sea and surrounding areas, i.e., the Anatolian and Arabic plates, are excluded from the analysis in this work. These regions will be analyzed separately because they include a significant number of micro plates, which each has a characteristic motion that differs from that of the Eurasian plate. In the Asian part, the Deccan plate is omitted because it does not belong to the Eurasian plate.

## Figures and Tables

**Table 1:** Eurasian plate motion parameters obtained after adding individually selected stations (given in Appendix A) that are not consistent with the Eurasian plate motion or are not located on the Eurasian plate to calculation scenario 1.

No.	Name of the Station and Geodetic Position	Φ [°]	Λ [°]	ω [°/Ma]
30 (scenario 1 - final solution)		54.87 ± 0.47	261.02 ± 0.50	0.2585 ± 0.0026
31	30 (from scenario 1) + Bilibino (BILI), B = 67°, L = 166°	53.06 ± 0.47	259.60 ± 0.51	0.2500 ± 0.0026
31	30 (from scenario 1) + Magadan (MAGO), B = 59°, L = 150°	53.66 ± 0.56	259.71 ± 0.57	0.2505 ± 0.0030
31	30 (from scenario 1) + Petropavlovsk (PETP), B = 52°, L = 158°	50.41 ± 1.48	254.19 ± 1.34	0.2335 ± 0.0066
31	30 (from scenario 1) + Teheran (TEHN), B = 35°, L = 51°	55.11 ± 0.83	261.40 ± 0.77	0.2551 ± 0.0038
31	30 (from scenario 1) + Kunming (KUNM), B = 24°, L = 102°	54.03 ± 0.65	260.04 ± 0.68	0.2502 ± 0.0036
31	30 (from scenario 1) + Lhasa (LHAS), B = 29°, L = 91°	57.63 ± 0.98	265.05 ± 1.17	0.2666 ± 0.0062
31	30 (from scenario 1) + Mizusawa (MIZU), B = 38°, L = 141°	50.20 ± 1.84	254.67 ± 1.67	0.2334 ± 0.0082
31	30 (from scenario 1) + Koganei (KGNI), B = 35°, L = 139°	52.23 ± 1.32	257.04 ± 1.39	0.2330 ± 0.0069
31	30 (from scenario 1) + Usuda (USUD), B = 35°, L = 138°	52.30 ± 1.27	257.10 ± 1.24	0.2336 ± 0.0062
31	30 (from scenario 1) + Tsukuba (TSKB), B = 35°, L=140°	51.39 ± 1.44	256.25 ± 1.37	0.2333 ± 0.0068
31	30 (from scenario 1) + Abashiri (P202), B = 44°, L = 144°	52.42 ± 1.26	256.35 ± 1.22	0.2340 ± 0.0057

**Table 2:** Comparison of the Eurasian plate motion parameters estimated in this paper with solutions derived by Drewes [45] and Larson et al. [8].

No.	Solution	Φ [°]	Λ [°]	ω [°/Ma]
1	APKIM2005 model [45]	53.4 ± 0.4	264.3 ± 0.5	0.259 ± 0.001
2	Larson et al. [8]	56.3	257.2	0.26
3	Solution given in this paper (based on 120 GNSS station positions)	54.81 ± 0.37	261.04 ± 0.48	0.2585 ± 0.0025

## Appendix A

**Table A1:** Computed plate motion parameters, and their errors, of the Eurasian plate for the GNSS network using the sequential method. Scenario 1 of the calculations.

No.	Name of the Station	Φ [°]	Λ [°]	ω [°/Ma]
2	Ny-Alesund+Bucarest	53.04 ± 2.64	262.45 ± 1.79	0.2659 ± 0.0183
3	2 + Paris	53.61 ± 2.35	261.37 ± 1.49	0.2643 ± 0.0151
4	3 + Yerevan	54.00 ± 1.90	262.84 ± 1.33	0.2642 ± 0.0136
5	4 + Simeiz	53.72 ± 1.69	261.46 ± 1.20	0.2637 ± 0.0109
6	5 + Copenhaguen	53.21 ± 1.45	261.73 ± 1.10	0.2609 ± 0.0098
7	6 + Uzhgorod	53.49 ± 1.28	262.01 ± 1.01	0.2635 ± 0.0077
8	7 + Graz	53.82 ± 1.10	262.22 ± 0.94	0.2634 ± 0.0070
9	8 + Borowiec	54.00 ± 0.97	262.09 ± 0.86	0.2627 ± 0.0067
10	9 + Venezia	54.06 ± 0.87	262.44 ± 0.81	0.2630 ± 0.0059
11	10 + Wettzell	54.28 ± 0.80	262.11 ± 0.78	0.2625 ± 0.0052
12	11 + Lviv	54.11 ± 0.77	261.94 ± 0.73	0.2621 ± 0.0048
13	12 + Sofia	54.35 ± 0.74	261.62 ± 0.71	0.2615 ± 0.0045
14	13 + Novossibirsk	54.21 ± 0.72	261.27 ± 0.69	0.2598 ± 0.0040
15	14 + Marseille	54.40 ± 0.68	261.36 ± 0.68	0.2597 ± 0.0040
16	15 + Shanghai	54.62 ± 0.67	261.49 ± 0.64	0.2594 ± 0.0038
17	16 + Pecny	54.73 ± 0.66	261.42 ± 0.63	0.2595 ± 0.0038
18	17 + Jozefoslaw	54.70 ± 0.63	261.24 ± 0.57	0.2593 ± 0.0037
19	18 + Domen	54.70 ± 0.60	261.20 ± 0.55	0.2590 ± 0.0035
20	19 + Ulan Bator	54.76 ± 0.60	261.15 ± 0.53	0.2589 ± 0.0033
21	20 + Perugia	54.78 ± 0.57	261.07 ± 0.53	0.2588 ± 0.0032
22	21 + Kitab	54.69 ± 0.57	261.10 ± 0.53	0.2587 ± 0.0031
23	22 + Grasse	54.63 ± 0.51	261.14 ± 0.53	0.2585 ± 0.0029
24	23 + Madrid Robledo	54.46 ± 0.50	261.25 ± 0.53	0.2585 ± 0.0028
25	24 + Zimmerwald	54.55 ± 0.48	261.30 ± 0.52	0.2585 ± 0.0028
26	25 + Istanbul	54.65 ± 0.48	261.18 ± 0.52	0.2583 ± 0.0027
27	26 + Klaipeda	54.70 ± 0.48	261.09 ± 0.52	0.2585 ± 0.0026
28	27 + Irkoutsk	54.84 ± 0.47	261.03 ± 0.51	0.2585 ± 0.0026
29	28 + Herstmonceux	54.90 ± 0.47	261.07 ± 0.50	0.2585 ± 0.0026
30	29 + Chumysh	**54.87 ± 0.47**	**261.02 ± 0.50**	**0.2585 ± 0.0026**

## Appendix B

**Table A2:** Computed plate motion parameters, and their errors, of the Eurasian plate for the GNSS network using the sequential method. Scenario 2 of the calculations.

No.	Name of the Station	Φ [°]	Λ [°]	ω [°/Ma]
2	Metsahovi+Matera	53.64 ± 2.75	262.33 ± 3.30	0.2644 ± 0.0259
3	2 + Trondheim	53.18 ± 2.25	261.88 ± 2.77	0.2621 ± 0.0176
4	3 + Potsdam	53.55 ± 1.99	261.65 ± 2.33	0.2593 ± 0.0136
5	4 + Toulouse	53.74 ± 1.54	261.34 ± 1.86	0.2577 ± 0.0123
6	5 + La Rochelle	54.13 ± 1.31	261.00 ± 1.55	0.2586 ± 0.0093
7	6 + Villafranca	54.88 ± 1.00	260.88 ± 1.23	0.2574 ± 0.0085
8	7 + Torino I	55.10 ± 0.86	260.93 ± 1.15	0.2601 ± 0.0080
9	8 + Changchun	55.32 ± 0.79	260.52 ± 1.10	0.2587 ± 0.0076
10	9 + Wuhan	55.06 ± 0.74	261.07 ± 1.02	0.2589 ± 0.0069
11	10 + Onsala	55.00 ± 0.71	261.43 ± 1.00	0.2582 ± 0.0063
12	11 + Vaasa	54.95 ± 0.66	261.05 ± 0.97	0.2585 ± 0.0059
13	12 + Penc	54.89 ± 0.62	260.83 ± 0.96	0.2581 ± 0.0054
14	13 + Wroclaw	54.79 ± 0.62	260.82 ± 0.96	0.2580 ± 0.0052
15	14 + Nicea	54.72 ± 0.60	260.96 ± 0.95	0.2582 ± 0.0048
16	15 + Chize	54.80 ± 0.54	260.92 ± 0.92	0.2587 ± 0.0045
17	16 + Boras	54.87 ± 0.53	261.03 ± 0.88	0.2596 ± 0.0044
18	17 + Stavanger	54.86 ± 0.50	261.20 ± 0.86	0.2590 ± 0.0042
19	18 + Mendeleevo	54.81 ± 0.49	261.14 ± 0.86	0.2594 ± 0.0040
20	19 + Port Vendres	54.84 ± 0.44	261.05 ± 0.81	0.2592 ± 0.0039
21	20 + Cagliari	54.86 ± 0.43	261.05 ± 0.72	0.2587 ± 0.0036
22	21 + Teddington	54.86 ± 0.42	260.95 ± 0.69	0.2585 ± 0.0036
23	22 + Roquetes	54.81 ± 0.41	260.92 ± 0.66	0.2586 ± 0.0034
24	23 + Cannes	54.81 ± 0.40	260.96 ± 0.64	0.2586 ± 0.0033
25	24 + Kootwijk	54.82 ± 0.40	261.01 ± 0.62	0.2585 ± 0.0031
26	25 + Terschelling	54.82 ± 0.39	260.97 ± 0.62	0.2584 ± 0.0031
27	26 + Westerbork	54.85 ± 0.39	261.01 ± 0.61	0.2585 ± 0.0029
28	27 + Braunschweig	54.83 ± 0.38	261.00 ± 0.59	0.2585 ± 0.0029
29	28 + Poltava	54.80 ± 0.38	261.02 ± 0.57	0.2585 ± 0.0029
30	29 + Tixi Seismic	**54.84 ± 0.38**	**260.98 ± 0.57**	**0.2585 ± 0.0029**

## Appendix C

**Table A3:** Computed plate motion parameters, and their errors, of the Eurasian plate for the GNSS network using the sequential method. Scenario 3 of the calculations.

No.	Name of the Station	Φ [°]	Λ [°]	ω [°/Ma]
2	Brest+Joensuu	51.62 ± 0.85	255.11 ± 0.98	0.2333 ± 0.0245
3	2 + Padova	53.26 ± 0.77	260.16 ± 0.92	0.2559 ± 0.0230
4	3 + Redu	53.45 ± 0.61	260.43 ± 0.74	0.2552 ± 0.0145
5	4 + Yebes	54.17 ± 0.60	260.87 ± 0.62	0.2552 ± 0.0140
6	5 + Wladyslawowo	54.30 ± 0.59	260.76 ± 0.58	0.2551 ± 0.0135
7	6 + Warnemuende	54.23 ± 0.59	261.01 ± 0.56	0.2564 ± 0.0130
8	7 + Sassnitz	54.52 ± 0.58	261.26 ± 0.55	0.2567 ± 0.0120
9	8 + Oberpfaffenhofe	54.52 ± 0.58	261.44 ± 0.53	0.2567 ± 0.0115
10	9 + Osan air base	54.63 ± 0.57	261.72 ± 0.52	0.2567 ± 0.0105
11	10 + Badary	54.77 ± 0.56	261.79 ± 0.52	0.2568 ± 0.0100
12	11 + Hsinchu	54.71 ± 0.56	261.61 ± 0.50	0.2568 ± 0.0095
13	12 + Zwenigorod	54.88 ± 0.55	261.40 ± 0.50	0.2572 ± 0.0090
14	13 + Daejeon	55.00 ± 0.55	261.45 ± 0.50	0.2571 ± 0.0073
15	14 + Suwon-Shi	54.81 ± 0.54	261.28 ± 0.49	0.2571 ± 0.0057
16	15 + Vilhelmina	54.66 ± 0.54	261.52 ± 0.49	0.2576 ± 0.0055
17	16 + Shaanxi	54.62 ± 0.53	261.34 ± 0.48	0.2577 ± 0.0040
18	17 + Beijng	54.80 ± 0.52	261.14 ± 0.48	0.2626 ± 0.0037
19	18 + Borkum	54.83 ± 0.49	261.17 ± 0.48	0.2626 ± 0.0035
20	19 + Helgoland Islan	54.79 ± 0.46	261.12 ± 0.48	0.2626 ± 0.0028
21	20 + Wabern	54.67 ± 0.43	261.06 ± 0.48	0.2626 ± 0.0021
22	21 + Cascais	54.78 ± 0.42	261.08 ± 0.47	0.2600 ± 0.0020
23	22 + Palma De mallor	54.80 ± 0.40	261.16 ± 0.47	0.2594 ± 0.0020
24	23 + Alicante	54.68 ± 0.39	261.07 ± 0.47	0.2585 ± 0.0019
25	24 + Morpeth	54.65 ± 0.38	261.05 ± 0.47	0.2585 ± 0.0019
26	25 + Brussels Ukkle	54.59 ± 0.37	260.99 ± 0.46	0.2585 ± 0.0018
27	26 + Moscow	54.56 ± 0.37	261.12 ± 0.46	0.2585 ± 0.0018
28	27 + Khabarovsk	54.56 ± 0.36	261.00 ± 0.46	0.2585 ± 0.0018
29	28 + Esbjerg	54.55 ± 0.36	261.07 ± 0.46	0.2585 ± 0.0018
30	29 + Krasnoyarsk	**54.56 ± 0.36**	**261.03 ± 0.46**	**0.2585 ± 0.0018**

## Appendix D

**Table A4:** Computed plate motion parameters, and their errors, of the Eurasian plate for the GNSS network using the sequential method. Scenario 4 of the calculations.

No.	Name of the Station	Φ [°]	Λ [°]	ω [°/Ma]
2	Ajaccio+Saint Jean des	55.09 ± 1.86	260.45 ± 2.06	0.2507 ± 0.0119
3	2 + Martsbo	55.30 ± 0.75	262.61 ± 1.29	0.2541 ± 0.0056
4	3 + Visby	55.56 ± 0.69	262.08 ± 1.25	0.2566 ± 0.0052
5	4 + Innsbruck Haf	55.22 ± 0.68	261.42 ± 1.19	0.2583 ± 0.0050
6	5 + Borowa Gora	55.03 ± 0.67	262.95 ± 1.12	0.2592 ± 0.0048
7	6 + Lamkowko	55.28 ± 0.64	262.20 ± 1.00	0.2610 ± 0.0044
8	7 + Riga	55.16 ± 0.62	261.73 ± 0.90	0.2622 ± 0.0040
9	8 + Kharkiv	54.99 ± 0.62	261.06 ± 0.84	0.2637 ± 0.0037
10	9 + Tashkent	54.83 ± 0.61	261.58 ± 0.70	0.2641 ± 0.0033
11	10 + Mikolajev	54.75 ± 0.61	261.35 ± 0.67	0.2646 ± 0.0033
12	11 + Svetloe	55.00 ± 0.61	261.15 ± 0.66	0.2654 ± 0.0033
13	12 + Zelenchukskaya	55.24 ± 0.61	261.49 ± 0.65	0.2585 ± 0.0032
14	13 + Redzikowo	55.15 ± 0.61	262.00 ± 0.65	0.2586 ± 0.0031
15	14 + Arti	55.00 ± 0.60	262.11 ± 0.65	0.2605 ± 0.0031
16	15 + Poligan Bishk	54.76 ± 0.60	262.05 ± 0.64	0.2615 ± 0.0031
17	16 + Morpeth	54.99 ± 0.58	261.84 ± 0.64	0.2611 ± 0.0030
18	17 + Genova	54.90 ± 0.57	261.40 ± 0.62	0.2602 ± 0.0029
19	18 + Lowestoft	54.78 ± 0.55	261.36 ± 0.61	0.2586 ± 0.0029
20	19 + North Shields	54.73 ± 0.55	261.31 ± 0.60	0.2594 ± 0.0028
21	20 + Ganovce	54.64 ± 0.51	261.27 ± 0.60	0.2594 ± 0.0027
22	21 + Vacov	54.54 ± 0.49	261.21 ± 0.59	0.2585 ± 0.0025
23	22 + Bellmunt de Seg	54.56 ± 0.47	261.33 ± 0.58	0.2585 ± 0.0025
24	23 + Cantabria	54.71 ± 0.46	261.35 ± 0.57	0.2586 ± 0.0024
25	24 + Valencia	54.60 ± 0.44	261.25 ± 0.55	0.2585 ± 0.0024
26	25 + Thessaloniki	54.52 ± 0.43	261.22 ± 0.54	0.2585 ± 0.0024
27	26 + Kiev	54.72 ± 0.42	261.35 ± 0.54	0.2585 ± 0.0024
28	27 + Liverpool	54.67 ± 0.42	261.23 ± 0.53	0.2585 ± 0.0024
29	28 + Norilsk	54.64 ± 0.42	261.29 ± 0.53	0.2585 ± 0.0024
30	29 + Wakkanai	**54.69 ± 0.41**	**261.22 ± 0.53**	**0.2585 ± 0.0024**
